# Delivery of Euthyroid Baby following Hyperthyroidism in Twin Gestation with Coexisting Complete Hydatidiform Mole

**DOI:** 10.1155/2019/2941501

**Published:** 2019-12-26

**Authors:** Rishi Raj, Edilfavia Mae Uy, Matthew Hager, Kamyar Asadipooya

**Affiliations:** Department of Endocrinology, Diabetes and Metabolism, University of Kentucky, Lexington, KY, USA

## Abstract

**Context:**

Gestational trophoblastic disease (GTD) is a rare complication of pregnancy, ranging from molar pregnancy to choriocarcinoma. Twin pregnancies with GTD and coexisting normal fetus are extremely rare with an estimated incidence of 1 case per 22,000–100,000 pregnancies. Molecular mimicry between human chorionic gonadotrophin (hCG) and thyroid-stimulating hormone (TSH) leads to gestational trophoblastic hyperthyroidism (GTH) which is further associated with increased maternal and fetal complications. This is the first reported case in literature describing the delivery of a baby with biochemical euthyroid status following a twin pregnancy with hydatidiform mole (HM) associated with gestational trophoblastic hyperthyroidism (GTH).

**Case Description:**

A 24-year-old G4 P3 Caucasian female with twin gestation was admitted to hospital for gestation trophoblastic hyperthyroidism. She was later diagnosed to have twin pregnancy with complete mole and coexisting normal fetus complicated by gestational trophoblastic hyperthyroidism (GTH). Despite the risk associated with the continuation of molar pregnancy, per patient request, pregnancy was continued till viability of the fetus. The patient underwent cesarean section due to worsening preeclampsia and delivered a euthyroid baby at the 24th week of gestation.

**Conclusions:**

Twin pregnancy with gestational trophoblastic disease and coexisting normal fetus is associated with high risk of hyperthyroidism, and careful monitoring of the thyroid function test along with dose titration of thionamides is of utmost importance throughout the gestation. If normal thyroid hormone levels are maintained during the pregnancy, euthyroidism could be successfully achieved in the baby.

## 1. Case Presentation

**Figure 1:**
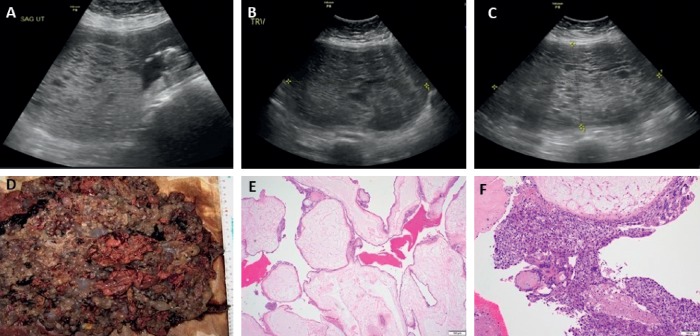
Ultrasound imaging of twin gestation (complete HM and coexisting normal fetus) at 14 weeks (a), 22 weeks (b), and 24 weeks (c). Histologic examination: (d) gross appearance with multiple tan-gray semitransparent vesicles of variable size admixed with solid fragments of tan-brown soft tissue and blood clot with no fetal parts; (e) diffuse villous enlargement with marked hydropic change; absent p57 immunostain (not shown) supporting the diagnosis of complete hydatidiform mole; (f) circumferential trophoblastic proliferation with focal necrosis and cytologic atypia, characteristics of complete HM.

A 24-year-old G4 P3 Caucasian female with a history of hypertension and type II diabetes mellitus presented to the emergency room with vaginal bleeding at 13 weeks of gestation. On presentation, she reported dizziness, diaphoresis, tremors, anxiety, palpitations, nausea, and leg swelling. Review of system was negative for headache or scotomas. Her vital signs showed blood pressure 141/80 mmHg, heart rate 97 beats per minute, respiratory rate 20 per minute, and temperature 98.4°F. Physical examination was significant for gravid uterus and bilateral lower extremity edema and had fine tremors of both hands. Thyroid gland was normal in size without any nodules. Laboratory studies (Tables 1 and 2) revealed suppressed TSH, elevated free T4, and absence of thyroid antibodies including thyrotropin-receptor antibodies (TRAb), thyroperoxidase antibodies (TPOAb), and thyroglobulin antibodies (TgAb). 24 hour urine protein was 200 mg/day and urine protein/creatinine ratio 0.2 in a random urine specimen. Pelvic ultrasound showed twin gestation with one complete HM and one coexisting normal fetus (Figures 1(a)–1(c)). Based on clinical and biochemical evidence of hyperthyroidism with elevated human chorionic gonadotrophin (hCG) levels in the absence of autoantibodies, a diagnosis of GTH was made.

**Figure 2:**
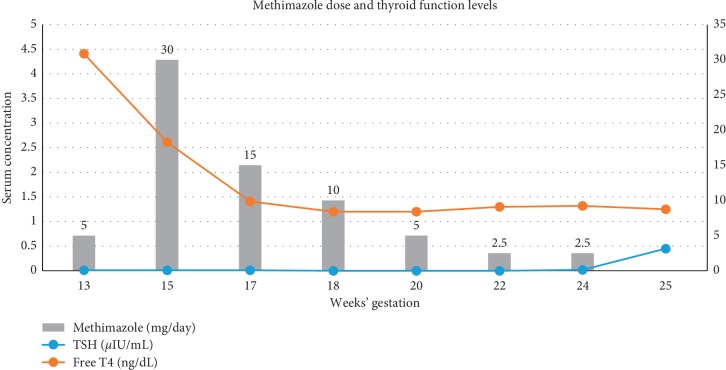


After extensive discussion regarding risk of developing complications associated with molar pregnancy [1], she preferred to proceed with her current pregnancy until viability of fetus. As she was into her 2^nd^ trimester of pregnancy, she was started on methimazole and the dose was titrated to achieve a goal free T4 in the upper range of normal (normal range = 0.8–1.7 ng/dL) to prevent fetal hypothyroidism. She was also on metoprolol 25 mg daily for chronic hypertension which was continued throughout the pregnancy. She also developed preeclampsia at 16 weeks of pregnancy with 24 hr urine protein of 3729 mg/day. She was again recommended termination of pregnancy, but she preferred to proceed with her pregnancy. At 24^th^ weeks of gestation, pregnancy was complicated by worsening preeclampsia and she eventually underwent a lower segment cesarean section with delivery of a viable fetus and evacuation of molar pregnancy. The weight of the baby was 645 gram, and APGAR score was 1 at 1 minute, 2 at 5 minutes, and 3 at 10 minutes. Postoperatively, gross examination of the tissue revealed fragments of pink-red spongy soft tissue without any fetal parts or abnormal Z line, while microscopic examination revealed hydropic villi and trophoblastic proliferation confirming the diagnosis of a complete mole (Figures 1(d)–1(f)). Histopathology of placenta revealed a singleton placenta weighing 156 grams and measuring 13.8 × 11.3 × 2.6 cm. At the time of delivery, she was on very low dose of methimazole, and therefore, it was discontinued. She remained clinically and biochemically euthyroid in the postpartum period. Her hCG gradually trended to a normal range of 17 weeks after delivery, and she had spontaneous remission of molar pregnancy as supported by low hCG levels at 1-year follow-up (Figure 2). The preterm infant did not have any gross abnormalities, and screening for congenital hypothyroidism was negative both at birth with TSH < 2.91 *μ*U/mL (normal range < 20 *μ*U/mL) and free T4 8.2 *μ*g/dL (normal range ≥ 5 *μ*g/dL) and at two months postdelivery with TSH < 2.91 *μ*U/mL (normal range < 20 *μ*U/mL) and free T4 6.7 *μ*g/dL (normal range ≥ 5°*μ*g/dL). Unfortunately, the baby needed mechanical ventilation for respiratory distress syndrome and gradually developed bronchopulmonary dysplasia requiring need for prolonged mechanical ventilation and underwent tracheostomy. He was transferred to a long-term care facility.

## 2. Discussion

Gestational trophoblastic disease (GTD) is defined as a spectrum of proliferative disorders of trophoblastic cells and includes benign nonneoplastic placental site trophoblastic tumor, HM (complete or partial), gestational trophoblastic neoplasia, and choriocarcinoma [2]. HM is the most common form of GTD and results from errors of fertilization. It can be classified into complete or partial mole on the basis of karyotype, gross morphology of the specimen, histopathologic features, and clinical features [2]. Extreme maternal age (≤15 and >35 years) and previous history of molar pregnancy are considered to be risk factors [3]. In the US, HM occurs between 0.5 and 2.5 per 1000 pregnancies [3]. Twin gestation with HM is even rarer with an estimated incidence of 1 per 20,000 to 100,000 pregnancies [4] and is associated with severe maternal complications (massive vaginal bleeding, hyperemesis gravidarum, hyperthyroidism, preterm delivery, pregnancy-induced hypertension, preeclampsia, trophoblastic embolization, and gestational trophoblastic neoplasia) and fetal complications (fetal death) [1, 4–6]. A literature review by Lin et al. revealed a regional difference in clinical presentation of CHMCF with a higher rate of life-threatening conditions in South America compared to North America [1].

In our case, the patient chose to pursue her pregnancy until her fetus becomes viable for delivery knowing the possibility of severe complications of her pregnancy. If HM is associated with viable fetus as in our case, it is important to distinguish the specific type of mole as the normal cotwin of the complete mole has a better survival rate compared to the partial mole [7]. Cytogenetic analysis of complete HM reveals a diploid karyotype without fetal parts and has an androgenetic origin, while the partial mole has a triploid karyotype with fetal parts [7]. The fetus in the partial mole has triploidy without chance of life after delivery, and therefore, partial mole even with fetus alive should be interrupted [8]. P57 immunohistochemical staining helps in distinguishing between a complete and partial mole as seen in our case which is absent in complete HM and present in partial HM [9].

hCG is a glycoprotein hormone, made of a common alpha subunit encoded by a single gene on chromosome 6 and a hormone specific beta subunit encoded by a cluster of genes on chromosome 19 [10]. hCG shares the same alpha subunit as well as significant amino acid sequence in the beta subunit with TSH and hence is able to interact with the TSH receptor [11]. However, its potency for TSH receptor is significantly (∼4000 times) less than TSH [12], and hence development of hyperthyroidism requires sustained elevation of hCG levels to >200,000 mIU/mL for several weeks [10]. In a normal pregnancy, secretion of hCG begins by the 3^rd^ week and peaks to 200,000–300,000 mIU/mL between 9 and 11^th^ weeks before gradual decline to 3,500–150,000 mIU/mL by the 20^th^ week [10]. The peak hCG lasts for only few days and can occasionally lead to transient thyrotoxicosis with resultant elevation of T3 and T4 levels and suppression of TSH, bearing a mirror image of the hCG peak; however; treatment is rarely required beyond the 22^nd^ week of pregnancy [12]. In contrast to normal pregnancy, HM secretes a large amount of hCG proportional to the tumor mass [12]. For every 10,000 mIU/ml increase in serum hCG, FT4 increases by 0.1 ng/dL and TSH decreases by 0.1 mIU/mL [13]. Furthermore, hCG produced from GTD is considered to have enhanced thyrotrophic activity compared to hCG in normal pregnancy as supported by increased cAMP production by GTD-associated hCG action on human TSH-R on Chinese hamster ovary cells [14]. This persistently elevated levels of hCG in molar pregnancy can lead hyperthyroidism and is defined as gestational trophoblastic hyperthyroidism.

Hyperthyroidism as a complication of HM was first described in 1955 [2]. The prevalence of biochemical hyperthyroidism with HM is relatively common and varies from 25 to 64% with 5% having clinical hyperthyroidism [10]. Clinically evident hyperthyroidism can manifest with weight loss, heat intolerance, palpitation, tremors, and diaphoresis with severity of symptoms being directly proportional to hCG levels [12]. In patients with uncontrolled hyperthyroidism, surgery and anesthesia may precipitate thyroid storm resulting in increased perioperative mortality [2].

Surgical evacuation of the molar pregnancy is the mainstay of management for HM and leads to prompt reduction in thyroid hormone levels secondary to decreased hCG concentrations [2]. In twin pregnancy with gestational trophoblastic disease and coexisting normal fetus, early termination of pregnancy had traditionally been advised but due to a better understanding of this condition, continuation of select pregnancy has been considered. Favorable obstetric outcome is seen among patients without antenatal maternal complications (pregnancy-induced hypertension, hyperthyroidism, and hyperemesis gravidarum) and initial serum hCG level less than 400,000°mIU/mL [4]. In those cases, continuation of twin pregnancy with gestational trophoblastic disease and coexisting normal fetus is an acceptable option with up to one-third chance of a live birth (7–37%) [7]. In our patient, although she developed preeclampsia and had significant hyperemesis gravidarum, she had successful fetal and maternal outcome which is extremely rare.

If pregnancy is desired, hyperthyroidism is mostly treated with beta blockers and thionamides while waiting for definitive management of the GTD [15]. The goal of thionamides is to reduce and maintain the mother's serum FT4 levels within a high normal range for nonpregnant females using the lowest dose to prevent fetal goiter and fetal hypothyroidism [15]. Transient central hypothyroidism may be seen in infants whose mother had poorly controlled hyperthyroidism during pregnancy, presumably due to suppression of the fetal-pituitary-thyroid-axis [15]. This is due to effect of uncontrolled maternal hyperthyroidism which leads to suppression of fetal TSH [16]. Both methimazole (MMI) and propylthiouracil (PTU) are equally effective in controlling hyperthyroidism. The choice of thionamide is based on consideration of their teratogenicity and risk of serious liver injury. The MMI embryopathy phenotype was described which includes scalp defects from aplasia cutis, choanal and esophageal atresia, trachea-esophageal fistula, abdominal wall defects like umbilicocele, developmental delay, athelia/hypothelia, hearing loss, and dysmorphic facie [10, 11]. American Thyroid Association (ATA) recommends use of PTU in the first trimester and MMI afterwards to reduce the risk of congenital malformations from MMI in the first trimester [15]. Beta adrenergic blocking agents (BBs) used for controlling hypermetabolic symptoms should be titrated based on clinical symptoms, as long-term use of BB has been associated with intrauterine growth restriction, fetal bradycardia, and neonatal hypoglycemia [15].

On extensive review of literature, we came across only one case of twin pregnancy with gestational trophoblastic disease and coexisting normal fetus where thyroid status of the newborn baby was discussed. In that case, the mother was treated with PTU and newborn delivered at 26 weeks had low FT4 and low normal TSH at birth but normalized at 5 weeks [11]. In our case, normal levels of FT4 were maintained during the course of pregnancy through a strict titration of MMI dose based on frequent monitoring of FT4 levels, and the baby was biochemically euthyroid on delivery as well as on follow-up, suggestive of importance of strict control of thyroid hormone levels. As the mother's hyperthyroidism was well controlled with antithyroid medication, this might have resulted in euthyroid status of the baby.

It is also important for postevacuation surveillance to detect persistence of molar tissue for potential development of gestational trophoblastic neoplasia (GTN) or choriocarcinoma [7]. Approximately, 3–5% of cases of HM can develop choriocarcinoma, whereas incidence of GTN following twin pregnancy with gestational trophoblastic disease and coexisting normal fetus is as high as 37% which is considerably higher compared to incidence of GTN with HM [4, 7, 10]. In our case, hCG levels started to trend down immediately postoperatively and was normal at 17 weeks postpartum. hCG levels are usually followed weekly until it reaches a normal range, which is usually within two to three months of evacuation of the molar pregnancy as seen in this case [17]. Once hCG levels are within the normal reference range, patients are followed every month for six months to look for any evidence of gestational trophoblastic neoplasia [2]. Intervention is not needed unless there is a rise of hCG levels [2, 17].

## 3. Conclusion

Twin pregnancy with gestational trophoblastic disease and coexisting normal fetus is an extremely rare condition, and there are limited data on safety outcome of mother and fetus. If pregnancy is desired until fetus becomes viable, careful monitoring of the mother for life-threatening complications could result in successful maternal and fetal outcome. As twin pregnancy with gestational trophoblastic disease and coexisting normal fetus is associated with high risk of hyperthyroidism, careful monitoring of the thyroid function test along with dose titration of thionamides is of utmost importance throughout the gestation, and if normal thyroid hormone levels are maintained during the pregnancy, euthyroidism could be safely achieved in the baby.

## Figures and Tables

**Table 1:** Laboratory evaluation on admission.

Laboratory test	Result	Reference range
Anti-thyroid peroxidase Ab	<5	0–9 IU/mL
T3	405	87–187 ng/dL
Thyroglobulin antibody	<1.0	<4.0 IU/mL
TSH receptor antibody	<0.90	≤1.75
Urine protein creatinine ratio	0.2	<0.2 mg/mg
Total protein/day, urine	200	<150 mg/day if ambulatory <80 mg/day if bed rest
Platelet count	135	155–369 k/*μ*L
Hemoglobin	9.1	11.2–15.7 g/dL
Hematocrit	27.9%	34–45%
WBC	4.9	3.7–10.3 k/*μ*L
Glucose	83	74–99 mg/dL
Creatinine	0.35	0.60–1.10 mg/dL
Sodium	139	136–145 mmol/L
Potassium	3.9	3.7–4.8 mmol/L
Alkaline phosphatase	33	35–104 U/L
Alanine transaminase	9	8–33 U/L
Aspartate transaminase	8	11–32 U/L
Total bilirubin	0.3	0.2–1.1 mg/dL
LDH	171	116–250 U/L

**Table 2:** TSH, free T4, and hCG trends during and postpregnancy.

Laboratory Studies	TSH (range = 0.4–4.2 *μ*IU/mL)	Free T4 (range = 0.8–1.7 ng/dL)	Methimazole dose (mg/day)	hCG, total beta (range <5 mIU/mL)
*Weeks of gestation*				
13^th^ week	0.01	4.4	5	480, 579
15^th^ week	0.01	2.6	30	746, 811
17^th^ week	0.01	1.4	15	771, 692
18^th^ week		1.2	10	706, 583
20^th^ week		1.2	5	655, 027
22^nd^ week		1.3	2.5	357, 387
24^th^ week	0.02	1.3	2.5	415, 666

*Postdelivery*				
0.5^th^ week	0.45	0.8		15, 942
2^nd^ week				585
4^th^ week				430
8^th^ week				119
13^th^ week				13
17^th^ week				3
21^st^ week				<1

## Abbreviations

HM: Hydatidiform mole
hCG: Human chorionic gonadotrophin
GTD: Gestational trophoblastic disease
GTN: Gestational trophoblastic neoplasia
MMI: Methimazole
PTU: Propylthiouracil
TSH: Thyroid-stimulating hormone
BB: Beta-blocker.
